# New products

**DOI:** 10.1155/S1463924690000359

**Published:** 1990


Journal of Automatic Chemistry, Vol. 12, No. 6 (November–December 1990), pp. 280–284

### New products

### Software for analytical data processing and display

ADEPT SCIENTIFIC have been appointed exclusive UK distributors for the software products developed by Galactic Industries of Salem, New Hampshire, USA. Galactic's successful product line includes software for laboratory data integration, acquisition, processing and networking. The latest package, Lab Calc is designed to provide a powerful method of handling the large amount of data generated by such analytical techniques as chromatography and spectroscopy.

Lab Calc is a fast, flexible package that provides capability to process, display and plot analytical data on a PC. The software is designed on 'open architecture' principles, making it compatible with most laboratory instruments. Data can be collected and processed direct from the instrument via serial communications or a network. The software supports up to eight separate channels, data from which may be viewed collectively or independently. The speed of acquisition easily copes with kinetics and real-time chromatography or spectroscopy. Alternatively, Lab Calc can be used off-line, working on data stored in DOS files, or with data collection continuing in the background.

The real-time graphics capability allows both two- and three-dimensional data to be viewed with a contour representation that may be zoomed, expanded and rescaled interactively. Up to 30 levels of contour lines may be displayed in up to eight colours. Multi-level, menu-driven operation makes Lab Calc easy to use, with a choice of pop-up windows or standard horizontal menus. Extensive on-line help is included.

The data processing capabilities of the package are extensive and include all standard number-crunching routines as well as less usual functions such as polynomial baseline subtraction and adjustment of raw chromatograms to observe run-to-run retention time differences. The software includes a large library of pre-programmed data processing applications, which, coupled with Galactic's complete scientific data manipulation language, provides the tools for a range of customized functions.

Lab Calc's multi-dimensional data-handling capacity enables the system to process the huge data sets generated by hyphenated techniques such as diode-array/LC, GC/IR and LC/IR, as well as 2D NMR. Chromatograms can be extracted from time-resolved, multi-spectral runs and spectra extracted from multi-detector chromatographic runs.

Multiple chromatograms or spectra can be overlaid for comparison, and both chromatographic and spectral data may be viewed simultaneously. Peak positions and edges, amplitudes, areas, percentage areas, concentrations and compound names can be shown, and peaks labelled with retention time, area and other peak table information.

A menu-driven method editor allows sophisticated chromatographic methods including instrument control functions to be constructed. These methods may incorporate fast peak picking and integration, timed events, baseline override, forced peaks, negative peaks and advanced auto-injector and pump control.

Enquiries to: *Valerie Wood, Adept Scientific Micro Systems, 6 Business Centre West, Avenue One, Letchworth, Hertfordshire SG6 2HB, UK. Tel.: 0462 480055.*

### Choosing the right thermometer

Temperature measurement related to quality control in the food industry is an important issue. In keeping with their continued commitment to provide the food industry with information on temperature measurement and to assist in the training of catering staff, Kane-May Ltd have produced a leaflet on *Choosing the Right Food Thermometer*. Regulations for hygiene and safety in the food industry and the impending Food Act in the UK specify temperatures for all aspects of catering. This new leaflet will help the purchasers to avoid expensive mistakes by removing the jargon from technical specifications and giving advice on using and maintaining a thermometer.

This latest Fact Sheet is part of a series of Food Fact Sheets which have been supported by the Institution of Environmental Health Officers. These are now used extensively throughout the food industry providing general 'do's and don'ts' for all levels of catering staff.

*Enquiries to: Tracey Gibson, Kane-May Ltd, Swallowfield, Welwyn Garden City, Hertfordshire AL7 1JP, UK. Tel.: 0707 331051; fax: 0707 331202.*

### Chromatography vial data-sheets

Chromacol has started publishing a series of data-sheets showing the compatibility of their chromatography vials with leading auto-sampler brands. The first two are available for the LDC Promis and Marathon, and also the Spark Promis and Marathon. Information is based on tests performed in the laboratory of either the respective instrument company (or one of the official dealers) using Chromacol's standard glass and plastic vials, as well as their new 'Gold' range. The necessary caps and seals are also described. The data-sheets include a reference chart on the compatibility of the seals with a range of typical chromatography solvents.

*Copies are free from Charlie Cook, Chromacol Ltd, Glen Ross House, Summers Row, London N12 0LD. Tel.: 081 368 7666; fax: 081 361 4698.*

### HPLC solutions for bioscience

*Chromatography Solutions for Bioscience* describes the separation of biological molecules performed on Hewlett-Packard's high performance liquid chromatography (HPLC) systems. The brochure (Publication 5952-0622) features more than 25 typical application problems facing biochemists and biological scientists today. The major areas covered are:

(1) Protein purification.
(2) Protein analysis.
(3) Peptide separation.
(4) Amino-acid analysis.
(5) Nucleotides and nucleic acids.
(6) Carbohydrates.
(7) Biochemical messengers (catecholamines).

A column selection guide included in the brochure lists suitable columns and explains why and how a particular material should be used. The brochure clarifies the misconception that only one class of columns—glass columns—is right for biological molecules such as proteins and peptides. A full list of HP columns in glass and stainless steel completes the guide.

The brochure documents many separations developed on HP instruments in industry and academia with a list of references to the literature.

*Enquiries to Verena Haller, Hewlett-Packard SA, 150 Route du Nant-d'Avril, CH-1217 Meyrin (GE) 2, Switzerland. Tel.: 41 22 780-8227.*

### Stirrers

The Scientific Division of Sartorius has announced a comprehensive range of IKAVISC measuring stirrers which will enable accurate viscosity and rheology measurements to be recorded. The current range of eight models and associated impellers makes the stirrers suitable for a wide variety of applications: paints and varnish, petrochemical, textile, paper, cellulose, pharmaceutical, gypsum type industries, water authorities and in the biotechnology field.

The IKAVISC measuring stirrers have been grouped into the MR Highly Precise range, which offers readouts from as low as 1Nm/Ncm to 200 Nm/Ncm, and the MR Universal range which has torque ratings from 100 Ncm up to 1200 Ncm. The actual viscosity measurement is determined by the type of impeller being used.

A feature of the IKAVISC equipment system have been combined into one unit. The twist in the torsion bar is used to convert into an actual torque readout through the electronic measuring system. The signals from the torque sensor are processed and a readout given on the large digital display.

In addition to a measuring mode, the versatility of the IKAVISC equipment is extended with its 'limit' alarm feature. Two alarm settings can be selected. An optimum 'limit' can be selected, as can an upper or lower value. When either the upper or lower limit is exceeded an alarm can be triggered. This alarm output, apart from giving an audible warning, can be used to trigger valves etc., in such applications as batching.

By offering the MR Highly Precise and MR Universal range of measuring stirrers, Sartorius is able to satisfy the requirements of both the laboratory and more arduous production areas.

*For further information contact Peter Butler, Sartorius Ltd, Scientific Division, Longmead Business Centre, Blenheim Road, Epsom, Surrey KT19 9QN, UK. Tel.: 03727 45811; fax: 03737 20799.*

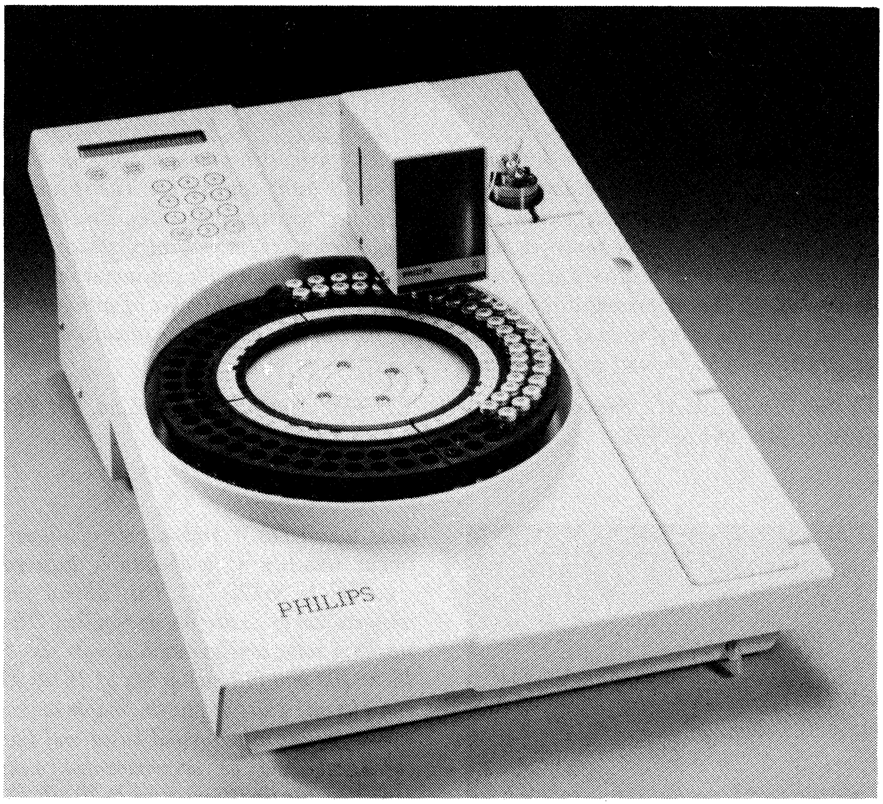

*Philips analytical Chromatography's PU4247 HPLC autosampler complements the company's range of modular, isocratic and gradient pumps. Key features of the autosampler are reproducible injection volumes with minimal carry-over, together with ease of use and reliability. Loop flushing is performed by head space pressure in combination with a metering, flow-through dispenser, making the system economic with sample volumes. Only flushed loop injections are used.*

*The pneumatic actuator system is dependable and fast, with a switching time of less than 50 ms. In addition, there is a fixed connection between the sample needle and injector, which means there is no troublesome combination of loop filler ports with septum needles.*

*The autosampler features an RS232 interface for data transfer to an integrator or laboratory computer. It also has inputs and outputs at TTL level for remote start/stop for next vial and injection, and a BCD output for the vial number.*

*Further information from Jane Cox, Philips Analytical Chromatography, York Street, Cambridge CB1 2PX, UK. Tel.: 0223 358866; fax: 0223 374542.*

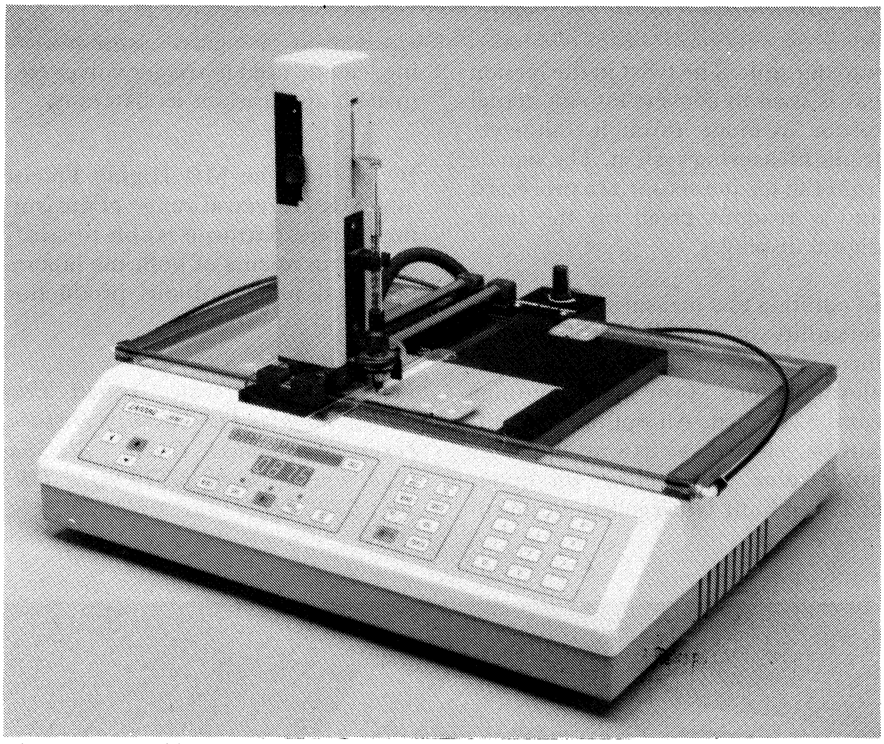

*An inert gas blanket for the Linomat IV protects oxidation-prone samples or their components against attack by atmospheric oxygen while being sprayed on. The stream of nitrogen propelling the sample will not, on its own, protect sensitive substances. By a kind of water-jet pump action, the spray jet sucks in air and hence oxygen molecules, which make intimate contact with the finely dispersed spray mist and can react with substances sensitive to oxidation. This is prevented if spraying on is effected under a blanket of nitrogen. In addition, during spraying on of successive samples, the starting zones of the samples already applied remain under inert gas protection.*

*Details from CAMAG, Sonnenmattstrasse 11, CH 4132 Muttenz, Switzerland. Tel. 061 613434; fax: 061 610702.*

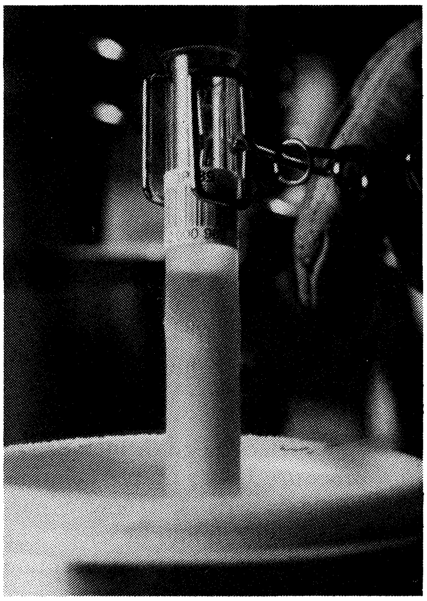

*The world's first photographic bar code label capable of adhering to frozen test-tubes has been launched by Computype. As it is almost impossible to stick directly to a frozen glass tube on which there is often condensation, the new label has an over-sized transparent seal which wraps back round on itself, hence allowing the bar code message still to be read whilst accurately and permanently identifying the test-tube. Advantages will include security and efficiency. Details from David Drinnan, Computype Ltd, International Information Centre, Compu/22, Oslo Road. Sutton Fields, Hull HU8 0YN, UK.*

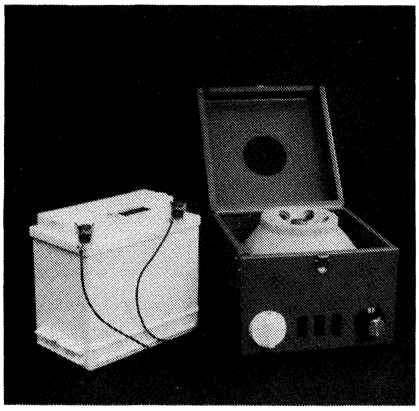

*Centrilab has developed the CEP 390-P Benchtop centrifuge to provide rugged performance from a 12V DC supply such as a car battery or portable generator. Intended for use where a mains supply is unrealiable or is not available the CEP 390-P is a simple, easy to operate unit with large manual controls and an exchangeable rotor.*

*Rugged component construction includes a steel housing and removable polypropylene rotor which has a high degree of dimensional stability at temperatures between −20 °C to +125 °C. This allows for refrigeration or sterilizing as required. The standard rotor configuration accepts six of 16 × 100 mm (12 ml) or six of 16 × 95 mm (10 ml) glass or plastic sample tubes. Custom rotors allow round based and flat based tubes to be accommodated with capacities ranging from 5 ml to 30 ml.*

*Details from Simon Ellis, Centrilab, Kingsbury House, Fridays Cross Mews, Christchurch Road, Ringwood, Hampshire BH24 1DG, UK. Tel.: 0425 480455.*

### Automatic dissolution

Dissolution testing is widely used in the pharmaceutical industry for formulation testing and as a quality-control parameter. Perkin-Elmer now offers a PC-controlled automatic system based on UV/Vis spectrometric detection of the active compounds. Sampling is done by parallel, continuous or intermittent sample flow from up to eight dissolution vessels in closed loops. The Perkin-Elmer Dissolution Software (PEDS) package controls the full system based on USP and GLP recommendations. Functions include spectrum scanning, multi-wavelength calibration, result manipulation and data export. All parameters are stored on disk as a parameter file and all raw data are automatically saved on disk for post-analysis evaluation. PEDS offers command chaining for optional automation of operations.

The Lambda 2 PC-controlled UV/Vis spectrometers are designed for routine and automated analyses. They offer total flexibility in method definition, double beam optics for long-term stability, basic methods for Scan, Timedrive, Wavelength Pro-

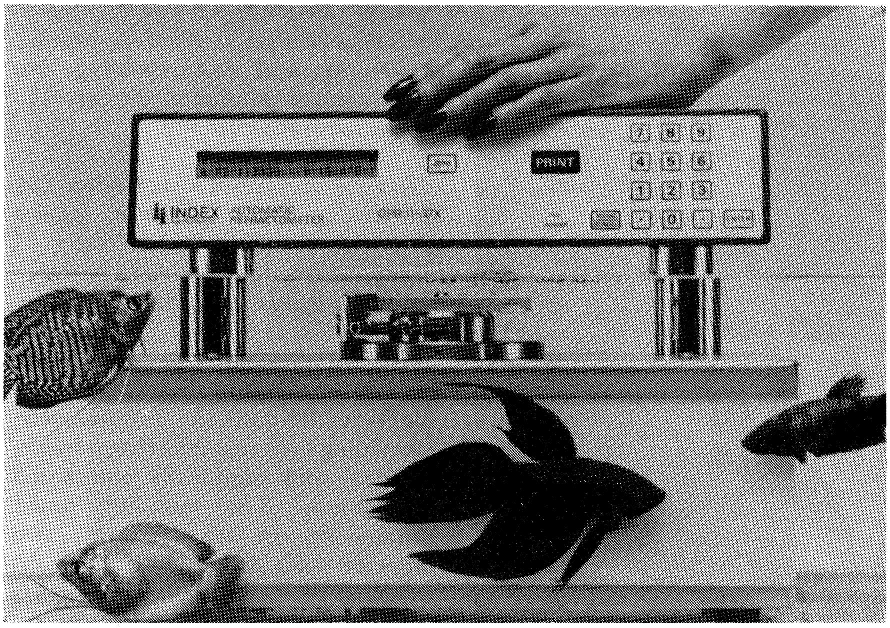

*Index Instruments has launched a more accurate general-purpose refractometer. The refractometer reads to 0.00005 Refractive Index Units (RI) or 0.05% sugar content (BRIX). The GPR11-37X is ideal for factory and quality control applications in many areas, for example sugar refining, the food industry, pharmaceuticals, chemicals and adhesives manufacture. Many of these are 'dirty' applications where the ability to douse the equipment with water during cleaning is of particular benefit. Readings are displayed on a 48-character LCD readout. There are also dual RS232 outputs for connection to a digital printer or computer. The GPR11-37X accommodates the full range of Index Instruments refractometer cells for bench or flow-through (on-line) process analysis.*

*Further information from Index Instruments Ltd, Bury Road Industrial Estate, Ramsey, Huntingdon, PE19 1NA, UK. Tel.: 0487 814313.*

gramme and Concentration and a wavelength range of 190 nm to 1100 nm.

*For further information contact Perkin-Elmer Ltd, Maxwell Road, Beaconsfield, Buckinghamshire HP9 1QA, UK. Tel.: 0494 676161; fax: 0494 678324.*

### Ultrasonic homogenizers

In order to facilitate such processes as microdisruption, emulsification and the production of suspension, the Biotechnology Division of B. Braun Medical Ltd has introduced the Labsonic series of competitively-priced ultrasonic homogenizers.

All Labsonic models have an output frequency of 20kH and have built-in timers for homogenization periods of between 0 and 15 min and various power outputs are available. For example, the Labsonic U is rated at over 340W, while the budget-priced labsonic L has an output in excess of 200W. B. Braun also supplies a comprehensive range of accessories for its homogenizers.

Labsonic homogenizers utilize a piezo-electric transducer in the probe assembly, which produces an extremely-high accoustic pressure within the process fluid, thereby causing cavitation.

*Details from B. Braun Medical, Braun House, 13–14 Farmborough Close, Aylesbury Vale Industrial Park, Stocklade, Aylesbury, Buckinghamshire HP20 1DQ, UK. Tel.: 0296 393900; fax: 0296 435714.*

### Development of 386-based applications

STSC International has expanded its range of APL programming products with the APL*Plus II Runtime Developer's Kit, which enables developers to expand their user base at no additional cost.

The APL*Plus II Developer's Kit, which comprises both the APL*Plus II application development system and a runtime system, allows unlimited usage of the runtime system, giving developers the ability to distribute an unlimited number of APL-based applications to as many applications sites as needed for the price of about five full systems. A Conversion Kit is available for developers who are already using the APL*Plus II system and just want a runtime licence to distribute their applications.

APL*Plus II is a complete second generation APL system and powerful application development tool. It is specifically designed for software applications which unleash the true power and capacity of 386-based PCs. The APL*Plus II system is particularly well-suited for applications designed to solve complex numeric problems because of its exceptional handling of tables and multi-dimensional arrays. APL's concise code enables developers to work much more productively—up to 10 times faster than with conventional programming languages like C, Fortran and Pascal.

APL*Plus II has no limit on the application workspace size up to the limit of the available memory. It is, for example, possible to have a 15Mbyte workspace in a machine with only 16Mbytes of RAM. There is also no limit on variable size up to the size of the workspace and no performance penalty for using large arrays. Mainframe data types such as 32-bit integers and 1-bit Booleans are also supported. In addition, the APL session manager is fully integrated with the full-screen editor.

The APL*Plus II Developer's Kit is compatible with all industry standard 386-based PCs including the IBM PS/2 Model 80 and Compaq 386. It requires a minimum of 2Mbytes of RAM and DOS 3.3 or above to run. Although not necessary, an 80387 or 80287 math co-processor is advisable.

The APL*Plus II Developer's Kit and Conversion Kit are priced from £4000 to £6000.

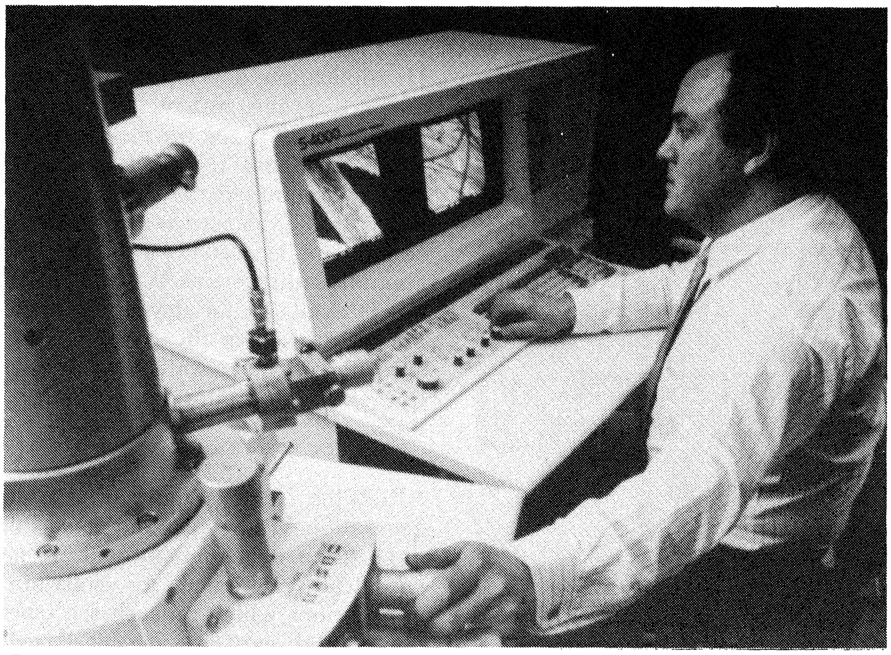

*Increased demand for sophisticated analytical and test facilities has led to the expansion of Focus Analytical, part of Courtaulds Research, based in Coventry, UK. Focus Analytical supplies a consultancy service from its laboratories within Courtaulds Research.*

*Services on offer range from service-led routine analysis to complex and detailed analytical investigations. Groups within Focus Analytical provide specialization in chromatography, thermal methods, nuclear magnetic resonance (NMR), spectroscopy, microscopy and elemental and chemical analysis for material characterization. Expertise in several of these techniques can be combined to resolve a problem.*

*For further information contact Norman Todd at Focus Analytical on 0203 688771, extension 2177.*

*Enquiries to: Suzy Wolfe, STSC International, Royal Albert House, Sheet Street, Windsor, Berkshire SL4 1BE, UK. Tel.: 0753 831451.*

### Centrifugal concentration and freeze drying

Genevac have introduced a combined centrifugal concentrator and freeze dryer—the SF50. Combined with the Cole vacuum pump—the CVP100, Genevac are able to offer an effective evaporation and freeze-drying system which operates without the need for chemical or cold traps.

This two-in-one laboratory evaporation system achieves rapid removal of aqueous or volatile organic solvents, acids and bases to dry or concentrate samples.

There are three versions of the SF50. The basic version has a heating and spin facility. The gauge version provides a bar graph display giving readings of vacuum pressure at the rotor chamber. The 'Vac-Stop' version includes a vacuum gauge and a controller. This allows the SF50 to be operated in a Vac-Stop mode in which the system shuts down automatically when the required level of dryness is reached.

A variety of rotors are available for the SF50 alternatively Genevac can provide custom rotors. A choice of three chambers are also available—standard acrylic, ported lyophilizer chamber with four ports able to accommodate four 250 ml flasks or safety borosilicate glass.

The chamber base plate and internal tubing are stainless steel for corrosion resistance and easy cleaning. No bearings are exposed to corrosives in the sample chamber.

*Details from Amanda Kerr, Genevac Ltd, 9 Farthing Road, Sproughton, Ipswich IP1 5AP, UK. Tel.: 0473 240000.*

### Built-in liquid autosampler

The AutoSystem Gas Chromatograph from Perkin-Elmer features a built-in 82-position autosampler, providing a cost-effective, space-efficient and completely automated instrument. The sampling tower rotates on an arc to access two injector ports, while allowing unobstructed access to the injection ports without removal of the autosampler.

Parameters for two separate automation modes can be programmed through the system keyboard, offering the ability to perform individual runs with different columns and conditions. The three AutoSystem speeds of injection and five GC methods are also programmed through the keyboard.

The two-line, 20-character vacuum fluorescence display shows the capillary column head-pressure. The patented packed-column flow readout is also displayed for accurate measurement. Addition of the optional OMEGA AutoSystem Controller allows simultaneous real-time monitoring and manipulation of instrument parameters and data for two AutoSystems.

Based on the OMEGA Chromatography Workstation which incorporates a mouse-pointing device, screen windows and dropdown menus for simplified operation, OMEGA AutoSystem Controller also provides unlimited GC method and autosampler program storage.

*For further information contact Perkin-Elmer Ltd, Maxwell Road, Beaconsfield, Buckinghamshire HP9 1QA, UK. Tel.: 0494 676161; fax: 0494 678324.*

